# Comparison of the Improvement Effect of Deep Ocean Water with Different Mineral Composition on the High Fat Diet-Induced Blood Lipid and Nonalcoholic Fatty Liver Disease in a Mouse Model

**DOI:** 10.3390/nu13051732

**Published:** 2021-05-20

**Authors:** Chung-Yu Lee, Chun-Lin Lee

**Affiliations:** Department of Life Science, National Taitung University, 369, Section 2, University Rd., Taitung 95092, Taiwan; pomalo3o@gmail.com

**Keywords:** deep ocean water, magnesium, calcium, potassium, nonalcoholic fatty liver disease

## Abstract

Accumulated lipid droplets in liver cause nonalcoholic fatty liver disease (NAFLD). Deep ocean water (DOW) containing high levels of magnesium, calcium, and potassium, etc. was proven to suppress hepatic lipid in obese rats fed high fat diet in the previous study. However, the effect of mineral compositions of DOW on the prevention of NAFLD is still unclear. This study removed calcium and potassium from DOW for modulating the mineral composition, and further compared the effects of DOW (D1(Mg + Ca + K)), DOW with low potassium (D2(Mg + Ca)), and DOW with low calcium and potassium (D3(Mg)) on the prevention of NAFLD in the mice model fed with high fat diet. In these results, DOW with high magnesium levels reduced serum and liver triglyceride and cholesterol levels and serum AST and ALT activities. However, when the calcium and/or potassium minerals were removed from DOW, the effects of reduction of triglyceride level, inhibition of acetyl-CoA carboxylase (ACC), fatty acid synthase (FAS), and peroxisome proliferator-activated receptor-alpha (PPAR-α) expressions, and activation of superoxide dismutase, catalase, and glutathione reductase activities would be weaker. In conclusion, DOW including magnesium, calcium and potassium minerals has the strongest preventive effect on NAFLD in a mouse model by increasing the antioxidant system and inhibiting fatty acid biosynthesis.

## 1. Introduction

The liver is an essential metabolic organ of the human body, and long-term liver damage causes liver cell injury. Combined with situations such as steatosis, fatty liver and liver inflammation may ultimately lead to liver cirrhosis, liver fibrosis, and even liver cancer. Obesity is a common health problem in developed countries and can incur chronic diseases, among which nonalcoholic fatty liver disease (NAFLD), including fatty liver and steatohepatitis [1,2], is one of the severest complications [3,4]. Over-intake of calories and fat causes lipids to accumulate in the liver, forming fatty liver, incurring lipid peroxidation, oxidative stress, and inflammation, gradually developing steatohepatitis, and finally leading to liver cirrhosis, fibrosis, and cancer [5].

By definition, deep ocean water (DOW) is the ocean water at 200 m below sea level that is characterized by low temperature, high stability, and containing abundant nutritive salts and micronutrients [6,7]. Due to the rich nutrients and low temperature characteristics of DOW, it is widely used in various industries, including aquaculture, skin health products, fermented foods, health foods, and medical research applications. [7,8,9]. Especially in health and medical research, DOW is focused on cardiovascular disease and metabolic syndrome in the applied research of medical care. Because of its rich mineral composition, deep ocean water has been pointed out to help improve hyperlipidemia, atherosclerosis, diabetes, obesity, and hypertension [10,11,12,13,14,15,16]. In a model where a high fat diet induced obesity in mice, DOW reduced weight gain and fat mass around the epididymis, as well as increased fatty acid oxidation and decreased fatty acid synthesis by regulating lipid synthesis and decomposition-related sterol regulatory element-binding protein 1c (SREBP1c), fatty acid synthase (FAS), and AMP-activate protein kinase (AMPK) mRNA expression levels, thus achieving an anti-obesity effect [12]. In a model where a high fat diet-induced obesity in hamsters, DOW reduced liver lipid accumulation by discharging lipids and bile acids through feces, and it increased fatty acid oxidation and decreased liver lipid accumulation by enhancing peroxisome proliferator-activated receptor-alpha (PPAR-α), retinoid X receptor alpha (RXR-α), and uncoupling protein-2 (UCP2) mRNA expression levels. In addition, DOW can decrease lipid peroxidation product (i.e., malondialdehyde (MDA)) levels and increase antioxidant enzyme (i.e., glutathione (GSH) and trolox equivalent antioxidant capacity (TEAC)) expression levels [17], thereby reducing liver injury caused by oxidative stress. A previous study has shown that a high fat diet can cause mitochondrial damage and reduce fatty acid oxidation and metabolism, thus exacerbating obesity, diabetes, and metabolic diseases [18]. DOW facilitates mitochondrial biogenesis and thus enhances fatty acid oxidation and metabolism and reduces the occurrence of obesity [19]

Research on minerals and blood lipids indicated that calcium and magnesium mineral supplementation can mitigate hypertension, reduce total cholesterol in serum, and inhibit cholesterol absorption by the small intestines by integrating divalent minerals and fatty acids to form insoluble matter [20]. After the induction of cholesterol generation in HepG2 cells and the addition of DOW containing 1500 ppm magnesium/calcium, DOW with a magnesium-to-calcium ratio of 3:1 can enhance pAMPK, whereas DOW with a magnesium-to-calcium ratio of 40:1 has no effect. Nevertheless, the two types of DOW can reduce cholesterol level [21].

Previous studies have demonstrated that DOW can prevent obesity, hyperlipidemia, and diabetes [10,12,13,14]. However, different types of DOW containing different compositions of minerals (produced by different water separation technology and concentration processes) may have different effects in mitigating NAFLD. Therefore, the effects of DOW types with different mineral compositions in mitigating NAFLD and the mineral-based regulation mechanism were explored to obtain the optimal composition of magnesium, calcium, and potassium minerals for mitigating NAFLD.

## 2. Materials and Methods

### 2.1. Chemicals

Ethanol (95%) was purchased from Taiwan Tobacco and Liquor Co. (Taipei, Taiwan). Gallic acid and Folin-Ciocalteau agent were purchased from Panreac Quimina S.A. (Barcelona, Spain).

### 2.2. The Source of DOW and Sample Preparation

DOW provided from the Eastern Taiwan Deep Sea Water Innovation and Research Center (Taitung, Taiwan) was pumped from a depth of 670 m in the Pacific Ocean near Eastern Taiwan and was processed as various DOWs with different mineral compositions through electrodeionization and vacuum concentration. In this study, three DOWs, including D1(Mg + Ca + K), D2(Mg + Ca), and D3(Mg), with the same hardness at 1400 ppm (according to the formula hardness (ppm) = Mg (mg/L) ∗ 4.1 + Ca (mg/L) ∗ 2.5) were used as the test substances in this study. D1(Mg + Ca + K) included 292.1 mg/L Mg, 81.0 mg/L Ca, 110.0 mg/L K, and 50.9 mg/L Na, D2(Mg + Ca) with low K concentration included 274.3 mg/L Mg, 110.1 mg/L Ca, 1.4 mg/L K, and 9.2 mg/L Na, D3(Mg) with low Ca and K concentrations included 341.3 mg/L Mg, 0.3 mg/L Ca, 1.8 mg/L K, and 4.1 mg/L Na. The DOW hardness was generally recommended to be 1400 ppm in the commercial product. Furthermore, the DOW with a hardness of 1400 ppm was also used as the substance for the hypercholesterolemic subjects in the previous clinical study [22]. Therefore, this animal dose is equivalent to drinking 500 mL DOW (hardness 1400 ppm) per day for a 60 kg adult. In order to decrease the amount of oral gavage for the animal, the three types of DOW (D1(Mg + Ca + K), D2(Mg + Ca) and D3(Mg)) with hardnesses 1400 ppm were respectively prepared as the animal substances of 10-fold DOW with hardnesses of 14,000 ppm by vacuum concentration.

### 2.3. Animals Diet

The formula diet was based on the commercial rodent diet product (Research diets Inc., New Brunswick, NJ, USA). The normal diet (3.85 kcal/g) included 18.96% casein, 0.28% L-cystine, 47.98% corn starch, 11.85% maltodextrin, 6.52% sucrose, 4.74% cellulose BW200, 2.37% soybean oil, 1.90% lard, 0.95% mineral mix, 1.23% dicalcium phosphate, 0.52% calcium carbonate, 1.56% potassium citrate, 0.95% vitamin mix V10001, 0.19% choline bitartrate. The high fat diet (4.74 kcal/g) included 23.31% casein, 0.35% L-cystine, 8.48% corn starch, 11.65% maltodextrin, 20.14% sucrose, 5.83% cellulose BW200, 2.91% soybean oil, 20.69% lard, 1.17% mineral mix, 1.51% dicalcium phosphate, 0.64% calcium carbonate, 1.92% potassium citrate, 1.17% vitamin mix V10001, 0.23% choline bitartrate). Regarding the mineral composition, the normal diet contained 0.135 mg/kg Mg, 1.354 mg/kg Ca, 0.975 mg/kg K, 0.271 mg/kg Na, and the high fat diet contained 0.166 mg/kg Mg, 1.665 mg/kg Ca, 1.199 mg/kg K, 0.333 mg/kg Na.

### 2.4. Animals Grouping and Experiment Schedule 

Forty male C57BL/6J mice at 6 weeks of age were purchased from the National Laboratory Animal Center (Taipei, Taiwan). The mice were kept in a temperature-controlled room (23 °C) under a 12L:12D cycle (light on at 6:00) and were given free access to regular rodent diet and water. The mice were randomly assigned to 5 groups of 8 animals (20 g of minimum body weight). This animal experiment was reviewed and approved by the Institutional Animal Care and Use Committee (IACUC) of the National Taitung University. 

The NOR group was daily fed normal diet for establishing the normal-based group. The NAFLD group was daily fed high fat diet in order to establish the negative control group with NAFLD. In order to compare the effects among the three types of DOW on the prevention of NAFLD, the DOW experiment groups including D1(Mg + Ca + K), D2(Mg + Ca), and D3(Mg) groups were fed the high fat diet daily and given different types but equal dose of 10-fold concentrated DOW daily (hardness 14,000 ppm; 10.25 mL/kg b.w) by oral gavage, respectively. Meanwhile, NOR and NAFLD groups were both daily given ultra-pure water by oral gavage. 

During the experiment, animal body weight and food intake were recorded. The minerals intake from diet can be calculated according to the mineral composition of diet and the feed intake content. The total minerals intake can be calculated from the sum of the minerals intake in the diet and that in the DOW. After 15 weeks, the mice were deprived of food for 16 h before being sacrificed by CO_2_ asphyxiation. Blood was collected from the posterior vena cava and centrifuged at 5000× *g* for 10 min; the serum was stored at −20 °C until analyzed. Liver tissue was collected and rinsed with 0.9% saline to remove excess blood. The second biggest liver tissue was isolated and immersed in 10% formaldehyde for histological inspection. The other liver tissues were stored at −80 °C for further analysis. 

### 2.5. Serum Biochemistry Parameters 

Serum triglyceride (TG) and total cholesterol(TC) were determined using the microplate spectrophotometer (Multiskan™ GO, Thermo Fisher Scientific Inc., Waltham, MA, USA) with triglyceride assay kit (BXC0271, Fortress Diagnostics Limited, Antrim, UK) and total cholesterol assay kit (BXC0271, Fortress Diagnostics Limited, Antrim, UK). Alanine transaminase (ALT) activity, aspartate aminotransferase (AST) activity, and alkaline phosphatase (ALP) were measured using an automatic clinical chemistry analyzer (Beckman-700, Fullerton, CA, USA).

### 2.6. Hepatic Lipid, TBARS and Anti-Oxidation Enzyme 

The liver tissue (0.1 g) were ground in 1 mL of ice-cold Folch solution (chloroform/methanol = 2:1; v/v) and incubated for 30 min at room temperature. The aqueous layer was aspirated and discarded, and the fixed volume of the organic layer was then evaporated to dryness. The dried lipid layer was dissolved with an equal volume of DMSO and then used to determine the TC and TG levels using commercial assay kit (BXC0271 and BXC027, Fortress Diagnostics Ltd., Antrim, UK).

The liver tissue was homogenized in lysis buffer (1% Triton X-100, 20 mM Tris, pH 7.5, 100 mM NaCl, 40 mM NaF, 0.2% SDS, 0.5% deoxycholate, 1 mM EDTA, 1 mM EGTA, and 1 mM Na_3_VO_4_) with a glass homogenizer and then centrifuged for 15 min at 12,000× *g* at 4 °C to obtain liver lysates. The lysates were used to evaluate lipid peroxidation by thiobarbituric acid reactive substance (TBARS) assay. 1,1,3,3-tetramethoxypropane was used as standard. Liver lysates (50 µL) were reacted with trichloroacetic acid (300 µL) and 60 mmol/L thiobarbituric acid (100 µL) and then were heated at 95 °C for 30 min. The mixtures were centrifuged for 20 min at 10,000× *g* and then measured at 532 nm [23].

For the determination of antioxidation enzyme activity, the liver tissue was homogenized in phosphate-buffered saline (1X-PBS, 0.026 M NaCl, 0.0026 M NaH_2_PO_4_, pH = 7). Superoxide dismutase (SOD) assay kit (SD 125, Randox, Crumlin, UK), catalase (CAT) assay kit (EnzyChromTM Catalase Assay Kit, ECAT-100, BioAssay Systems, Hayward, CA, USA), glutathione peroxidase (GPx) assay kit (RS 505, Randox, Crumlin, UK), and glutathione reductase (GRd) assay kit (GR 2368, Randox, Crumlin, UK) were used to determine the antioxidative enzymes.

### 2.7. Determination of Protein Expression 

The commercial ELISA kits including mouse PPAR-α ELISA kit (EM0479, Wuhan Fine Biotech Co., Ltd., Wuhan, China), mouse AMPK ELISA (EM0830, Wuhan Fine Biotech Co., Ltd.), mouse FASN ELISA kit (EM0611), mouse ACC1 ELISA kit (EK3132, Signalway Antibody, College Park, MD, USA), mouse SREBP-1 ELISA kit (MBS750834, MyBioSource, Inc., San Diego, CA, USA) were used to determine the protein expressions of PPAR-α, AMPK, FAS, acetyl-CoA carboxylase (ACC), and sterol regulatory element-binding protein-1 (SREBP-1).

### 2.8. Statistical Analysis 

Data are expressed as means ± standard deviation. The statistical one-way analysis of variance (ANOVA) was determined by using SPSS version 12.0 software (SPSS Institute, Inc., Chicago, IL, USA) with Duncan’s multiple test. Differences with *p* < 0.05 were considered statistically significant.

## 3. Results

### 3.1. The Contents of Magnesium, Calcium and Potassium Minerals Intake from the Sample and Diets

Table 1 shows the total amount of the main minerals magnesium, calcium and potassium minerals taken by the experimental animals from DOW sample and diet during the test period. The minerals in the animal diet provided the daily basic mineral requirements for the mice. The normal diet supported 3.85 kcal/g and high fat diet supported 4.74 kcal/g, therefore, the high fat diet has higher feed calories than the normal diet. In Table 1, A high fat and high-calorie diet led to lower food intake, therefore, the food intake of the NAFLD group was lower than that of the NOR group. Even though a high fat diet has a higher proportion of minerals than a normal diet, the high fat diet reduces food intake and leads to a decrease in mineral intake. The intake of NAFLD group was significantly lower than that of NOR group in magnesium, calcium, and potassium minerals contents. However, after daily supplementation of various DOW, the intake of three minerals in D1(Mg + Ca + K) group increased. The total amount of magnesium, calcium and potassium minerals reached 147.63 mg, 473.29 mg, and 351.20 mg, respectively. D2(Mg + Ca) group contains lower potassium mineral, so the total intake of potassium minerals is 319.32 1 mg, which is lower than D1(Mg + Ca + K) group. D3(Mg) group contains higher magnesium levels and lower calcium and potassium levels, and the total intake for 15 weeks were 454.57 mg and 327.84 mg, respectively. Except for the higher intake of magnesium minerals, the intake of other minerals was similar to that of the NAFLD group

### 3.2. Body Weight and the Ratio of Liver Weight to Body Weight 

A high fat diet can cause a large amount of triglycerides (TGs) to accumulate in the liver, thereby increasing liver weight [24]. The ratio of liver weight to body weight indicates whether a high TG level is present in the liver. However, the previous studies found no significant difference between a high fat diet and a controlled diet regarding the ratio of liver weight to body weight in the obese mice model induced by high fat diet (*p*  >  0.05) [25,26]. As Table 2 indicates, the liver weight of the NAFLD group was significantly higher than that of the NOR group (*p* < 0.05). However, no significant difference was observed between the NOR and NAFLD groups regarding the ratio of liver weight to body weight (*p*  >  0.05), which is consistent with the aforementioned studies. Nevertheless, compared with the NAFLD group, the ratios of liver weight to body weight for the D1(Mg + Ca + K), D2(Mg + Ca), and D3(Mg) groups were significantly decreased (*p* < 0.05). The results indicate that intake of DOW can reduce high fat diet-induced liver weight.

### 3.3. Aspartate Aminotransferase and Alanine Aminotransferase Activity Levels and Blood Urea Nitrogen Concentration Level in Serum

The enhancement of aspartate amnotransferase (AST) and alanine aminotransferase (ALT) activity levels in serum is a crucial indicator of liver injury [27,28]. According to the data in Table 3, AST and ALT activity levels in serum from the NAFLD group were significantly higher than those from the NOR group (*p* < 0.05). This result indicates that a high fat diet causes liver cell injury. Intake of DOW with different compositions of minerals can reduce AST and ALT activity levels. DOW with a lack of calcium and potassium can also significantly reduce AST and ALT activity levels enhanced by a high fat diet. Therefore, DOW with abundant Mg minerals can reduce the indices for liver functions whose values have risen because of a high fat diet. To ensure that the intake of DOW that contains a high level of minerals does not cause kidney injury, the blood urea nitrogen (BUN) level in serum, an index for kidney injury, was analyzed. Table 3 presents the results: no significant differences in the BUN level were observed in any groups (*p* > 0.05). Intake of DOW at the indicated level does not cause kidney injury.

### 3.4. Influence of Lipid Concentration in Serum 

A high fat diet can increase TG and TC levels in serum and the liver and can cause lipids to accumulate in the liver, forming fatty liver [5,29]. Table 4 indicates that TG and TC levels in serum from the NAFLD group were significantly higher than those from the NOR group (*p* < 0.05). A high fat diet can cause a high serum TG levels in an animal model. Intake of DOW containing magnesium, calcium, and potassium by the D1(Mg + Ca + K) group significantly reduced TG and TC levels in serum (*p* < 0.05). For the D2(Mg + Ca) and D3(Mg) groups, only the TG level in serum reduced, but the reduction was not significant (*p* > 0.05).

### 3.5. Lipid Concentration in the Liver 

Analysis result of liver lipids in Table 5 indicated that TG and TC levels in the livers of the NAFLD group significantly increased (*p* < 0.05) but decreased after being fed with DOW. The D1(Mg + Ca + K) group fed with a ratio of magnesium to calcium to potassium of 3:1:1 exhibited the most significant reduction in TG and TC levels in the liver (*p* < 0.05). This result indicates that DOW with a composition of magnesium, calcium, and potassium most effectively reduces lipid concentrations in serum and lipid accumulation in the liver. 

### 3.6. Lipid Peroxidation in the Liver 

**Figure 1:**
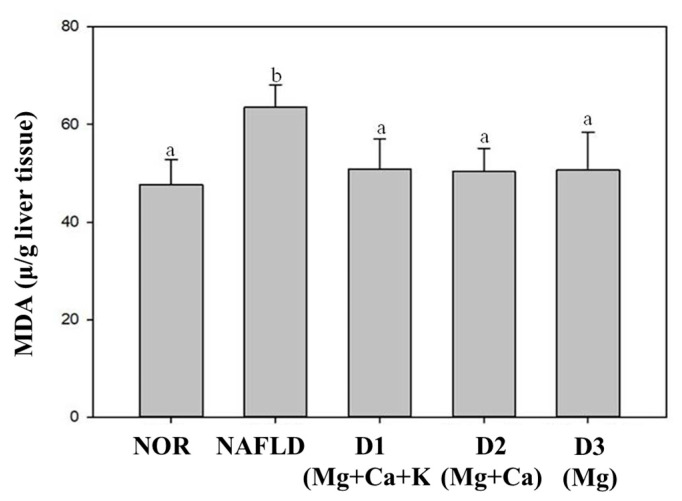
Effect of different mineral composition of deep ocean water constitutions on the lipid peroxidation in NAFLD mouse. Two groups of mice were administrated with normal diet (NOR group) or high fat diet (NAFLD group). Other high fat diet mice were administrated with deep ocean water (10.25 mL/kg/day, D1(Mg + Ca + K) group), low potassium deep ocean water (10.25 mL/kg/day, D2(Mg + Ca) group), low calcium and potassium deep ocean water (10.25 mL/kg/day, D3(Mg) group). The data are presented as the means ± SD (*n* = 8). Mean values with different superscripts (a and, b) are significant difference (*p* < 0.05). MDA: malondialdehyde.

Too much lipids accumulations increase oxidation and produce peroxides, which leads to lipid peroxidation in liver [24]. MDA content can be used as an indicator of the degree of lipid peroxidation in the body [30]. The results of this study are shown in Figure 1. Compared with the NOR group, the MDA content in the liver of the NAFLD group mice was significantly increased after 15 weeks of feeding a high fat diet (*p* < 0.05). It shows that a high fat diet can increase oxidative stress, but the three types of deep ocean water with high magnesium concentration can significantly reduce MDA content. Therefore, deep ocean water has the potential to reduce the oxidative stress caused by liver lipid peroxidation.

### 3.7. Activity of Antioxidative Enzyme in the Liver 

The evolution of fatty liver into fatty hepatitis is closely related to oxidative stress. Excessive lipids that accumulate in the liver can increase β-oxidation and electron transport chain activity; subsequently, electrons and oxygen molecules can form superoxide radicals. When the antioxidative enzyme in the body is insufficient, peroxides can harm cells and cause cell injury, thus incurring a series of oxidative inflammation responses [31]. The present study analyzed the activity levels of antioxidative enzymes in the liver (i.e., SOD, CAT, GPx, and GRd) to explore the antioxidative effect of DOW on high fat diet-induced NAFLD in mice.

**Figure 2:**
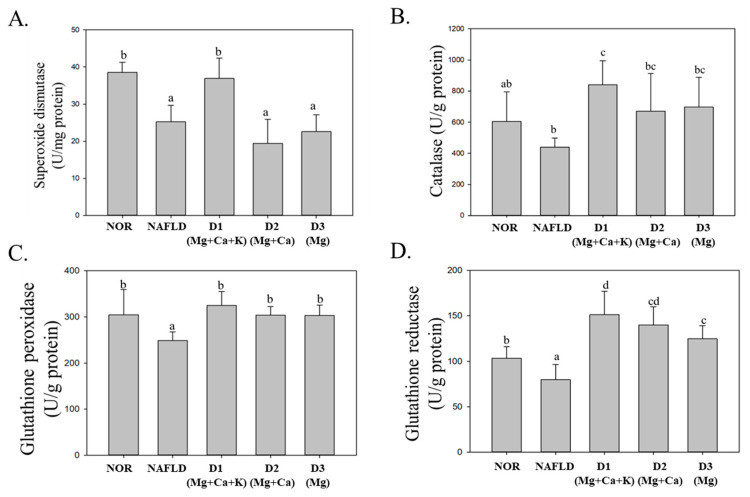
Effect of different mineral composition of deep ocean water constitutions on the activities of superoxide dismutase (**A**), catalase (**B**), glutathione peroxidase (**C**) and glutathione reductase (**D**) in NAFLD mouse. Two groups of mice were administrated with normal diet (NOR group) or high fat diet (NAFLD group). Other high fat diet mice were administrated with deep ocean water (10.25 mL/kg/day, D1(Mg + Ca + K) group), low potassium deep ocean water (10.25 mL/kg/day, D2(Mg + Ca) group), low calcium and potassium deep ocean water (10.25 mL/kg/day, D3(Mg) group). The data are presented as the means ± SD (*n* = 8). Mean values with different superscripts (a,b,c,d) are significant difference (*p* < 0.05).

As Figure 2 indicates, compared with the NOR group, the activity levels of SOD and CAT enzymes in the NAFLD group livers were reduced. This result implies that a high fat diet can reduce SOD and CAT activity levels. Compared with the NAFLD group, after being fed with DOW, SOD activity in only the D1(Mg + Ca + K) group significantly increased (*p* < 0.05). For the D2(Mg + Ca) and D3(Mg) groups, SOD activity reduced by the high fat diet was not significantly increased (*p* > 0.05). However, three types of DOW significantly increased CAT activity. The results indicate that DOW with low potassium and/or calcium minerals failed to increase SOD activity but increased CAT activity. 

GPx and GRd are both the antioxidative enzymes in GSH antioxidative system. GPx can decompose H_2_O_2_ through glutathione (GSH) oxidation, thereby avoiding the forming of oxidative stress [32]. GRd can react with nicotinamide adenine dinucleotide phosphate (NADPH) to reduce oxidized glutathione (GSSG) into GSH. The present results suggest that compared with the NOR group, GPx and GRd activity levels in the NAFLD group were significantly reduced (*p* < 0.05), whereas GPx and GRd activity in those fed with DOW containing different minerals and a high level of magnesium significantly increased (*p* < 0.05). This result indicates that the magnesium minerals in DOW are critical for increasing GPx and GRd activity levels. In addition, the GRd activity in the D1(Mg + Ca + K) group substantially increased because they were fed with DOW containing calcium and potassium minerals, thereby enhancing the activity of antioxidative enzymes reduced because of the high fat diet. 

### 3.8. Biopsy of Liver Tissues 

**Figure 3:**
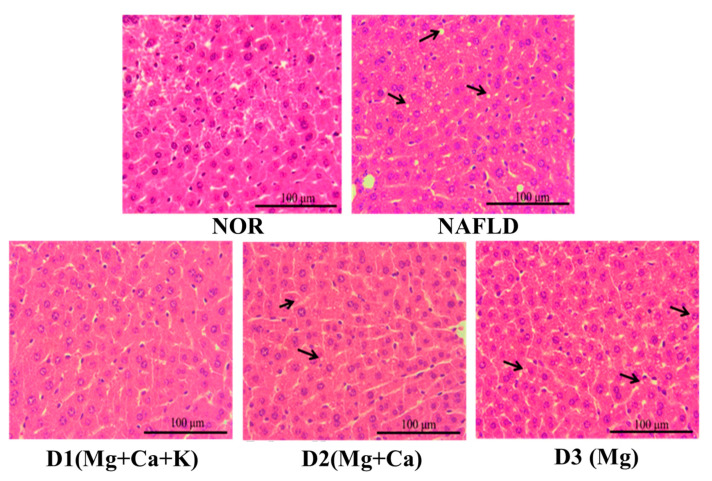
The effects of different mineral composition of deep ocean water on hepatic pathological changes of high fat diet-induced liver injury mice (400×). Two groups of mice were administrated with normal diet (NOR group) or high fat diet (NAFLD group). Other high fat diet mice were administrated with deep ocean water (10.25 mL/kg/day, D1(Mg + Ca + K) group), low potassium deep ocean water (10.25 mL/kg/day, D2(Mg + Ca) group), low calcium and potassium deep ocean water (10.25 mL/kg/day, D3(Mg) group). Representation images of H&E stained section after NAFLD and the treatment by deep ocean water. The black arrows indicated fat vacuoles of microvesicula and microvesicular types.

C57BL/6J male mice were fed a high fat diet for 15 weeks and then sacrificed. Subsequently, their livers were removed and processed with H&E dye to assess lipid accumulation in their livers. Figure 3 presents the results. The arrow indicates fat vacuoles in the liver. For the NOR group, no fat vacuoles were present in liver cells. By contrast, the NAFLD group fed with a high fat diet presented numerous fat vacuoles. Feeding with the test substance D1(Mg + Ca + K) can effectively reduce fat vacuole and lipid accumulation in liver cells. A lack of potassium minerals and/or calcium minerals (e.g., as with the D2(Mg + Ca) and D3(Mg) groups) failed to reduce lipid accumulation. Therefore, potassium and calcium minerals in DOW can effectively reduce lipid accumulation. 

### 3.9. Lipid Metabolism and Biosynthesis-Related Protein Expression 

The liver regulates numerous factors related to lipid synthesis and metabolism, such as PPAR-α, AMP-activated protein kinase (AMPK), sterol regulatory element-binding protein (SREBP), FAS, and ACC. Over-intake of calories and fat can cause substantial TG accumulation in the liver, resulting in abnormal lipid metabolism, inhibiting fatty acid oxidation, increasing fat formation, and finally leading to NAFLD [33,34]. PPAR-α and AMPK are factors in lipid decomposition that facilitate decomposition by enhancing fatty acid oxidation and β-oxidation effects, regulating long-chain acyl-CoA synthetase genes, and reducing TG content [35].

**Figure 4:**
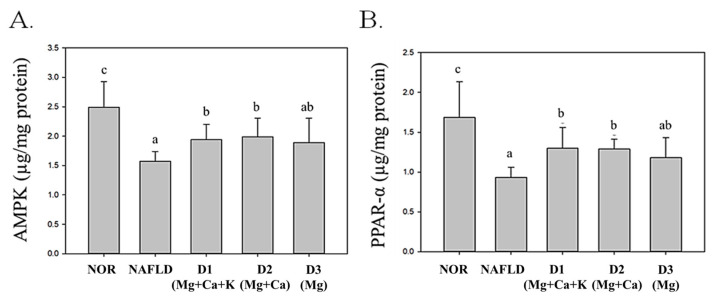
The effects of different mineral composition of deep ocean water on AMPK (**A**) and PPAR-α (**B**) of high fat diet-induced liver injury mice. Two groups of mice were administrated with normal diet (NOR group) or high fat diet (NAFLD group). Other high fat diet mice were administrated with deep ocean water (10.25 mL/kg/day, D1(Mg + Ca + K) group), low potassium deep ocean water (10.25 mL/kg/day, D2(Mg + Ca) group), low calcium and potassium deep ocean water (10.25 mL/kg/day, D3(Mg) group). The data are presented as the means ± SD (*n* = 8). Mean values with different superscripts (a,b,c) are significant difference (*p* < 0.05). AMPK: AMP-activate protein kinase, PPAR-α: proliferator-activated receptor-alpha.

As shown in Figure 4, because of its high fat diet, the NAFLD group’s PPAR-α and AMPK expression levels significantly decreased (*p* < 0.05); drinking DOW containing magnesium and calcium (D1(Mg + Ca + K) and D2(Mg + Ca) groups) significantly increased PPAR-α and AMPK expression levels in the liver (*p* < 0.05). However, drinking DOW with a lack of calcium and potassium minerals (the D3(Mg) group) failed to increase AMPK and PPAR-α expression levels, thus, fatty acid oxidation was reduced and TG accumulation in the liver increased. 

**Figure 5:**
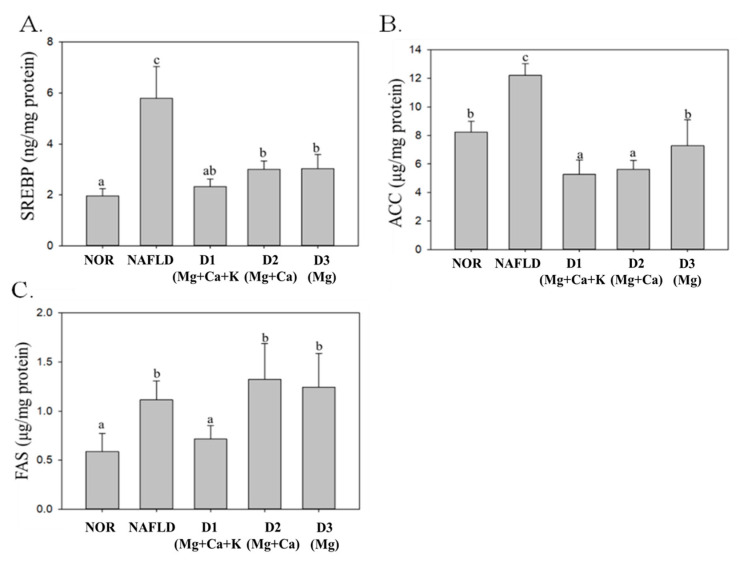
The effects of different mineral composition of deep ocean water on SREBP (**A**), FAS (**B**) and ACC (**C**) of high fat diet-induced liver injury mice. Two groups of mice were administrated with normal diet (NOR group) or high fat diet (NAFLD group). Other high fat diet mice were administrated with deep ocean water (10.25 mL/kg/day, D1(Mg + Ca + K) group), low potassium deep ocean water (10.25 mL/kg/day, D2(Mg + Ca) group), low calcium and potassium deep ocean water (10.25 mL/kg/day, D3(Mg) group). The data are presented as the means ± SD (*n* = 8). Mean values with different superscripts (a,b,c) are significant difference (*p* < 0.05). SREBP: sterol regulatory element-binding protein, FAS: fatty acid synthase, and ACC: Acetyl-CoA carboxylase.

SREBP, FAS, and ACC are factors in lipid synthesis that facilitate fatty acid synthesis and further convert free fatty acid into TGs to be stored in the liver [34]. As shown in Figure 5, because of the high fat diet, SREBP, FAS, and ACC expression levels in the NAFLD group significantly increased (*p* < 0.05). The SREBP, FAS, and ACC expression levels of D1(Mg + Ca + K) group significantly decreased (*p* < 0.05). However, D2(Mg + Ca) with low potassium levels and D3(Mg) with low calcium and potassium levels were both able to significantly decrease SREBP and ACC expression levels (*p* < 0.05) rather than FAS expression levels (*p* < 0.05). 

According to these results, DOW can regulate proteins related to lipid metabolism (i.e., PPAR-α, AMPK, SREBP, and ACC) due to magnesium. Both D2(Mg + Ca) and D3(Mg) containing magnesium and calcium minerals can increase PPAR-α and AMPK expression levels and reduce SREBP and ACC expression levels. However, D1(Mg + Ca + K) containing magnesium, calcium, and potassium minerals can have a further effect on repressing FAS expression levels. This result indicates that a complex composition of magnesium, calcium, and potassium minerals in DOW has the optimal effect on increasing lipid metabolism and reducing lipid synthesis.

## 4. Discussion

According to previous studies, a high fat diet was a suitable method to induce the animal model of NAFLD in C57BL/6J mice. A high fat diet not only increased the weight, index for liver injury (AST), and TG content in serum and liver significantly but also caused lipid droplet in liver [36,37]. After induction of NAFLD rat model with a high fat diet, the activity levels of antioxidative enzymes (SOD and GPx) significantly decreased (*p* < 0.05) and lipid peroxide (MDA) levels significantly increased (*p* < 0.05) [38]. The results from the present study accord with the aforementioned studies where NAFLD was successfully induced in mice. In the present animal study, the induction mechanism was using high fat feed to enhance SREBP-1, ACC, and FAS expression levels in the liver, thereby increasing fatty acid synthesis and reducing AMPK and PPAR-α expression levels, thereby reducing fatty acid β-oxidation. Accordingly, lipid accumulation in the liver led to significantly increased TG in the serum and the liver and finally successfully inducing NAFLD.

DOW is regarded as a nature ocean resource due to the character of low temperature, high clean, and rich minerals [6,7]. The rich and complex composition minerals are difficult to be made by chemical addition. Therefore, DOW has been developed as healthy drinking water in Japan, Korea, Taiwan and the United States due to the complex and unique minerals composition. The hardness of the commercial DOW drink is generally recommended to be 1400 ppm, and 500–1000 mL is for adult per day. Furthermore, the high magnesium DOW with a hardness of 1410 ppm was also proven to have a hypolipidemic effect and liver lipid-lowering effect in the hypercholesterolemic subjects in the previous clinical study [22]. The clinical study also used the DOW pumped from the Pacific Ocean near East Taiwan, and modulated hardness to 1410 ppm. The high magnesium DOW (395 mg/L Mg, 35.7 mg/L Ca, 6.58 mg/L, K, and 33.1 mg/L Na) of the clinical study [22] has a similar mineral composition with the D3(Mg) (341.3 mg/L Mg, 0.3 mg/L Ca, 1.8 mg/L K, and 4.1 mg/L Na) of this present study, although there was some variation at the calcium levels. The high magnesium DOW also significantly reduced serum TC and MDA levels in hypercholesterolemic subjects [22]. In this study, D3(Mg) also had a potent effect on lowering the serum TC levels and liver TC and MDA levels, therefore, the hypolipidemic result in this animal study matched the previous clinical study. The roles of the calcium and potassium in DOW on the lipid metabolism were not investigated in the previous study. However, the mineral composition was an important factor for developing DOW functional food. 

DOW was also proven to improve atherosclerosis, dermatitis, diabetes, obesity, hyperlipidemia, and hypertension in the various animal models due to the rich minerals composition [10,11,12,13,14,15,16]. However, the mineral composition and mineral-mineral interaction should influence the health function of DOW. Magnesium has always been considered the main functional mineral in DOW, but calcium, potassium, and other trace elements are also important nutrient minerals for human health. This study modulated the DOW mineral composition by remaining magnesium and removing calcium mineral or calcium and potassium minerals, and further studied the effect on NAFLD prevention. In this study, the mice fed high fat diet were given 10.25 mL/kg 10-fold concentrated DOW orally (hardness 14,000 ppm), which was equivalent to an adult drinking 500 mL DOW daily (hardness 1400 ppm). Three DOW types with various mineral compositions on magnesium, calcium, and potassium minerals were used as a mineral supplement to investigate the effects on NAFLD prevention. 

**Figure 6:**
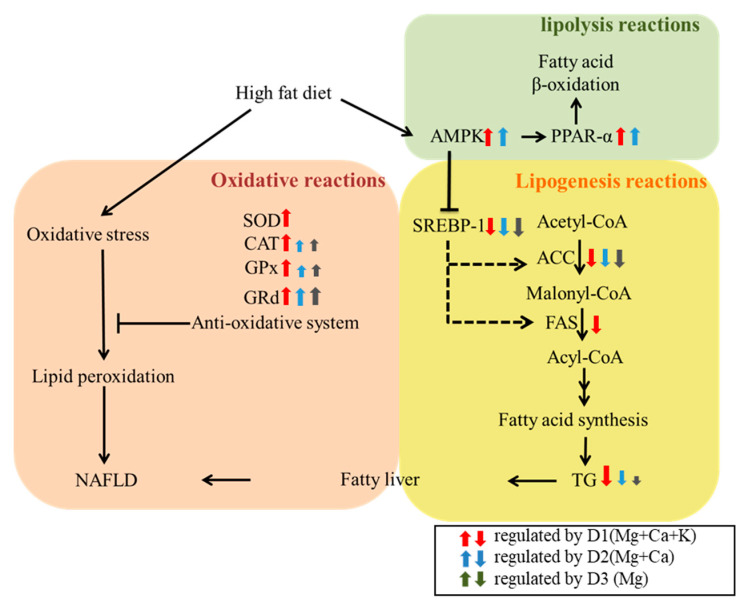
The metabolism and antioxidative regulations of various DOW with different mineral compositions in the high fat diet-induced NAFLD mice. NAFLD: nonalcoholic fatty liver disease, SOD: superoxide dismutase, CAT: catalase GPx: glutathione peroxidase, Grd: glutathione reductase. AMPK: AMP-activate protein kinase, PPAR-α: proliferator-activated receptor-alpha, SREBP: sterol regulatory element-binding protein, ACC: Acetyl-CoA carboxylase, and FAS: fatty acid synthase, TG: Triglyceride.

According to the results of this study (Table 1), the intake of magnesium, calcium and potassium minerals will be significantly reduced due to long-term high fat diet. After 15 weeks of long-term DOW supplementation, the total magnesium intake of group D1(Mg + Ca + K) was 102.82 mg more than that of NAFLD group. The common characteristic of D1(Mg + Ca + K), D2(Mg + Ca) and D3(Mg) is that they all contain high amount of magnesium minerals, and the hardness is 1400 ppm. However, their effects on the improvement of NAFLD were still different. Comparing the total intake of minerals by animals, the total potassium intake of D1(Mg + Ca + K) group was 18.72 mg more than that of D2(Mg + Ca) group. The calcium and potassium minerals intake of D1(Mg + Ca + K) group were 31.88 mg and 23.36 mg more than that of D3(Mg) group. Figure 6 presents that various DOW minerals compositions had different effects on modulating the lipid metabolism and antioxidative system. Although the increased calcium and potassium contents were less than 10% of the total intake. However, it can be seen from the results that D1(Mg + Ca + K) had a significant effect on lowering liver lipid levels, raising antioxidative system (SOD, CAT, GPX, Grd activities), raising lipolysis reactions (AMPK, PPAR-α expressions), and suppressing lipogenesis (SREBP-1, ACC, FAS expressions) in the liver due to its rich magnesium, calcium, and potassium minerals concentrations. D2 (Mg + Ca) with lower potassium minerals also had a prevention potential in the NAFLD mice model, but some indicators were decreased such as lowering liver TG, raising SOD and CAT activities, and suppressing FAS expression. However, D3 (Mg) had the weakest effect on NAFLD prevention. It had a weak effect on lowering liver TG, raising SOD and CAT activities, and had no effect on suppressing FAS expression and raising lipolysis factors expression (AMPK, PPAR-α). According to the above results, calcium and potassium minerals can have a synergistic effect with magnesium in DOW for improving NAFLD in a mice model.

Research confirmed that DOW could lower body fat in the obese rat induced by high fat diet for 8 weeks [29]. However, the present study explored the effects of different compositions of three minerals in mitigating NAFLD in the mice fed high fat diet for 15 weeks. In the result of Table 2, DOW can reduce liver weight and liver weight to body weight ratio, but it has no significant effect on reducing body weight. This may be because they are different animal models and experiment periods. In the past study, anti-obesity animal tests for DOW used rats as the model animal, and the test period with high- fat diet was only 8 weeks [29]. However, the mice were used as the model animal and fed high fat diet for 15 weeks in this study. In current, it is not clear from animal experiments that 15 weeks of long-term consumption of DOW has an effect on improving body weight raised by a high fat diet. Furthermore, the mineral composition and dosage of DOW samples were also different. This study used mice fed high fat diet as the animal model for inducing NAFLD. Mice model is more suitable for the study of NAFLD, because they can form more lipid droplets in the liver [39,40,41]. Therefore, it can be seen that the differences in animal species, test period, and test substances may affect the translation and application of experimental models. In addition to the reason for the differences, DOW supplementation did not influence the intake of food in this study. Therefore, DOW test groups and the NAFLD group should have similar total calorie intake and weight gain. That should be one of the reasons why DOW was difficult to reduce weight gain. Nonetheless, DOW was still proven to reduce liver weight and the ratio of liver weight to body weight in the NAFLD mice fed high fat diet. However, it should be further explored whether long-term drinking of DOW can maintain the weight loss effect of obese rat models in the future.

Table 3 and Figure 2 present comparisons of the effects of DOW with different mineral compositions in mitigating liver injury and increasing antioxidative system. DOW containing only magnesium minerals but lacking calcium and potassium minerals reduced AST (the index for liver injury), ALT activity, and MDA content (lipid peroxides). However, DOW that contained magnesium, calcium, and potassium minerals also had the optimal effect on enhancing the activity levels of antioxidative enzymes (SOD, CAT, and GPx). Previous studies have shown that feeding 250 mg/kg magnesium minerals to rats with diabetes enhanced total glutathione levels and reduced glutathione activity in the rats [42]. Therefore, magnesium minerals in DOW can enhance antioxidative enzyme activity. The present study determined that DOW with a lack of potassium minerals reduced the activity of antioxidative enzymes. A study has discovered that a lack of potassium minerals causes nitric oxide content in endothelial cells to decrease, resulting in endothelial dysfunction and then glucose intolerance and insulin resistance [43]. In sum, DOW containing magnesium minerals can reduce AST and ALT and thus protect the liver. Nevertheless, the synergetic effect of magnesium and potassium minerals is required to enhance the function of antioxidative enzymes in the liver and thus indirectly increase antioxidative enzyme activity, thereby mitigating liver injury due to oxidative free radicals.

This study compared the effects of DOW with different mineral compositions in reducing lipid content and improving lipid metabolism. DOW with high levels of magnesium, calcium, and potassium minerals (D1(Mg + Ca + K) group) had the strongest effect on improving NAFLD by decreasing TC and TG in serum and liver, reducing FAS, ACC, and SREBP-1 in the liver, and increasing fatty acid oxidation. D1(Mg + Ca + K) even had more effect on lowering liver TC and TG than the mice fed a normal diet. The reason may be that D1(Mg + Ca + K) had a strong effect on suppressing fatty acid biosynthesis via suppressing ACC expression in liver. In this study, DOW containing high magnesium still has a weak effect on improving lipid metabolism when it lacks calcium and/or potassium. DOW with low potassium and/or calcium minerals levels (D1(Mg) and D1(Mg + Ca)) failed to reduce TG content in the serum and the liver. DOW with low calcium and potassium minerals levels (D1(Mg)) was difficult to reverse AMPK and PPAR-α expression levels and promote fatty acid β-oxidation. DOW with low potassium and/or calcium minerals levels (D1(Mg) and D1(Mg + Ca)) were difficult to repress FAS expression levels and lipid synthesis. A study has demonstrated that feeding 100 mg/kg magnesium sulfide to rats with diabetes is conducive to the decrease of TG and cholesterol contents in serum [44]. In aP2-agouti transgenic mice, substantial amounts of calcium minerals can move into fat cells and therein increase FAS expression levels and reduce UCP2 expression levels, resulting in obesity. However, calcium minerals in a diet can reduce calcium mineral content in fat cells and both stimulate fat to decompose and inhibit fat formation, thereby decreasing weight and fat mass [45]. The present study determined that DOW lacking calcium minerals reduced AMPK and PPAR-α expression levels and then weaken fatty acid β-oxidation; accordingly, different types of DOW with different mineral compositions have differing effects on mitigating NAFLD. The results suggest that DOW with magnesium can reduce TG and TC levels in serum. In addition, the synergy between calcium and potassium minerals and magnesium minerals can enhance fatty acid β-oxidation and, thereby mitigating NAFLD.

The health function of DOW should contribute to the interaction among magnesium, calcium, potassium and other DOW minerals. Therefore, this study is not to explore the improvement of NAFLD by magnesium, calcium, and potassium alone, but to study the role of magnesium, calcium, and potassium in DOW and their interaction with each other. As shown in Figure 6, the regulation of DOW for liver lipids, antioxidant enzymes, and lipolysis and lipogenesis proteins expressions varies with different mineral compositions. D3(Mg) with high magnesium minerals and low calcium and potassium can regulate antioxidative enzyme (CAT, GPx, and GRd) activities levels and reduce fatty acid synthesis–related protein (SREBP and ACC) expression levels. The synergic effect between magnesium minerals and calcium minerals in D2(Mg + Ca) can further enhance β-oxidation and AMPK and PPAR-α expression levels, thereby increasing fatty acid β-oxidation and decreasing TG accumulation in the liver. D1(Mg + Ca + K) containing magnesium, calcium, and potassium has the biggest effect on the improvement of NAFLD. D1(Mg + Ca + K) can both effectively enhance antioxidative enzyme (SOD, CAT, GPx, and GRd) activities and increase fatty acid β-oxidation–related protein (AMPK and PPAR-α) expression levels as well as reduce fatty acid synthesis—related protein (FAS) expression levels, thus increasing fatty acid oxidation and decreasing lipid synthesis and then achieving the optimal effect in mitigating NAFLD. 

Regarding the limitation of this study, it is limited by the control of the mineral composition, so that the calcium or potassium was difficult to be removed from the DOW. The DOW of D3(Mg) still retains a small amount of calcium and potassium. In addition, the animal test is based on the load limit of the experiment scale, so only male animals were used in this experiment. However, the formation of fat and fatty liver in female animals may be different from male animals due to the influence of hormones. Furthermore, this study has not yet explored the effects of DOW dosage at low, medium and high doses on the prevention of NAFLD. The dose-dependent response of test substance is also important research. This study first aims to explore the difference in the improvement effect of DOW mineral composition on NAFLD. The above related limitations will be further studied in the future.

In conclusion, regarding the role of magnesium, calcium and potassium in DOW on the prevention of NAFLD, calcium minerals in DOW can influence fatty acid oxidation in the liver. DOW that contains potassium minerals may facilitate the enhancement of antioxidative enzyme activity. Magnesium minerals in DOW can reduce fatty acid synthesis–related protein expression levels. DOW with a ratio of magnesium minerals, calcium minerals, and potassium minerals of 3:1:1 had an optimal synergistic effect on the prevention of NAFLD. This research can provide a reference for the development of DOW beverages or mineral supplement beverages in the future.

## Figures and Tables

**Table 1:** The food intake and the contents of magnesium, calcium and potassium minerals intake from the sample and diets during the 15 weeks animal test.

Groups	Food Intake (g/Mouse)	Minerals Intake from the Sample (mg/Mouse)				Minerals Intake from the Diet (mg/Mouse)				Total Minerals Intake (mg/Mouse)			
		Mg^2+^	Ca^2+^	K^+^	Na^+^	Mg^2+^	Ca^2+^	K^+^	Na^+^	Mg^2+^	Ca^2+^	K^+^	Na^+^
NOR	363	-	-	-	-	49.2	492	354	98.4	49.2	492	354	98.4
NAFLD	269	-	-	-	-	44.8	448	322	89.6	44.8	448	323	89.6
D1(Mg + Ca + K)	268	103	27.0	38.8	17.9	44.6	446	321	89.3	148	473	360	107
D2(Mg + Ca)	266	96	36.5	0.49	3.22	44.3	443	319	88.6	140	479	319	91.8
D3(Mg)	273	118	0.10	0.63	1.42	45.5	454	327	90.9	164	455	328	92.3

Two groups of mice were administrated with normal diet (NOR group) or high fat diet (NAFLD group). Other high fat diet mice were administrated with deep ocean water (10.25 mL/kg/day, D1(Mg + Ca + K) group), low potassium deep ocean water (10.25 mL/kg/day, D2(Mg + Ca) group), low calcium and potassium deep ocean water (10.25 mL/kg/day, D3(Mg) group). The data are presented as the means ± SD (*n* = 8).

**Table 2:** Effect of different mineral composition of deep ocean water on body weight, liver weight and the ratio of liver to body weight in NAFLD mouse.

Groups	Initial Body Weight (g)	15 Week Body Weight (g)	Liver Weight (g)	Liver Weight/Body Weight (%)
NOR	23.4 ± 0.7 ^a^	24.6 ± 1.7 ^a^	0.83 ± 0.08 ^a^	3.37 ± 0.14 ^b^
NAFLD	23.0 ± 0.8 ^a^	29.9 ± 1.7 ^b^	0.97 ± 0.05 ^c^	3.26 ± 0.17 ^b^
D1(Mg + Ca + K)	23.6 ± 0.5 ^a^	29.9 ± 1.7 ^b^	0.88 ± 0.02 ^ab^	2.95 ± 0.11 ^a^
D2(Mg + Ca)	23.3 ± 0.9 ^a^	30.6 ± 2.4 ^b^	0.90 ± 0.05 ^b^	2.96 ± 0.07 ^a^
D3(Mg)	23.4 ± 1.3 ^a^	30.4 ± 2.3 ^b^	0.91 ± 0.08 ^b^	3.00 ± 0.14 ^a^

Two groups of mice were administrated with normal diet (NOR group) or high fat diet (NAFLD group). Other high fat diet mice were administrated with deep ocean water (10.25 mL/kg/day, D1(Mg + Ca + K) group), low potassium deep ocean water (10.25 mL/kg/day, D2(Mg + Ca) group), low calcium and potassium deep ocean water (10.25 mL/kg/day, D3(Mg) group). The data are presented as the means ± SD (*n* = 8). Mean values with different superscripts (a,b,c) are significant difference (*p* < 0.05).

**Table 3:** Effect of different mineral composition of deep ocean water on hepatic index AST and ALT in NAFLD mouse serum.

Groups	AST Activity (U/L)	ALT Activity (U/L)
NOR	105 ± 25 ^a^	47.9 ± 21.6 ^a^
NAFLD	198 ± 66 ^b^	88.2 ± 38.3 ^b^
D1(Mg + Ca + K)	105± 17 ^a^	34.5 ± 4.1 ^a^
D2(Mg + Ca)	128 ± 31 ^a^	46.0 ± 4.4 ^a^
D3(Mg)	106 ± 25 ^a^	35.1 ± 6.0 ^a^

Two groups of mice were administrated with normal diet (NOR group) or high fat diet (NAFLD group). Other high fat diet mice were administrated with deep ocean water (10.25 mL/kg/day, D1(Mg + Ca + K) group), low potassium deep ocean water (10.25 mL/kg/day, D2(Mg + Ca) group), low calcium and potassium deep ocean water (10.25 mL/kg/day, D3(Mg) group). The data are presented as the means ± SD *(n* = 8). Mean values with different superscripts (a and b) are significant difference (*p* < 0.05). AST: aspartate aminotransferase, ALT: alanine aminotransferase.

**Table 4:** Effect of different mineral composition of deep ocean water on serum TG, TC, and BUN levels in NAFLD mouse.

Groups	TG (mg/dL)	TC (mg/dL)	BUN (mg/dL)
NOR	31.5 ± 10.7 ^a^	85.1 ± 3.0 ^a^	24.0 ± 4.4 ^a^
NAFLD	76.0 ± 18.1 ^c^	121.2 ± 7.2 ^c^	24.9 ± 1.4 ^a^
D1(Mg + Ca + K)	55.1 ± 9.1 ^b^	101.2 ± 11.3 ^b^	22.9 ± 1.8 ^a^
D2(Mg + Ca)	68.5 ± 11 ^bc^	112.4 ± 11.0 ^c^	23.0 ± 2.8 ^a^
D3(Mg)	67.5 ± 16.3 ^bc^	101.4 ± 15.1 ^b^	23.9 ± 3.4 ^a^

Two groups of mice were administrated with normal diet (NOR group) or high fat diet (NAFLD group). Other high fat diet mice were administrated with deep ocean water (10.25 mL/kg/day, D1(Mg + Ca + K) group), low potassium deep ocean water (10.25 mL/kg/day, D2(Mg + Ca) group), low calcium and potassium deep ocean water (10.25 mL/kg/day, D3(Mg) group). The data are presented as the means ± SD (*n* = 8). Mean values with different superscripts (a,b,c) are significant difference (*p* < 0.05). TG: triglyceride, TC: total cholesterol, BUN: blood urea nitrogen.

**Table 5:** Effect of different mineral composition of deep ocean water on liver TG and TC levels in NAFLD mouse.

Groups	Liver TG (mg/g)	Liver TC (mg/g)
NOR	4.5 ± 0.6 ^ab^	1.9 ± 0.2 ^b^
NAFLD	5.6 ± 0.8 ^c^	2.4 ± 0.4 ^c^
D1(Mg + Ca + K)	3.9 ± 0.7 ^a^	1.6 ± 0.2 ^a^
D2(Mg + Ca)	4.2 ± 0.4 ^ab^	1.6 ± 0.3 ^ab^
D3(Mg)	4.9 ± 1.1 ^bc^	1.5 ± 0.2 ^a^

Two groups of mice were administrated with normal diet (NOR group) or high fat diet (NAFLD group). Other high fat diet mice were administrated with deep ocean water (10.25 mL/kg/day, D1(Mg + Ca + K) group), low potassium deep ocean water (10.25 mL/kg/day, D2(Mg + Ca) group), low calcium and potassium deep ocean water (10.25 mL/kg/day, D3(Mg) group). The data are presented as the means ± SD (*n* = 8). Mean values with different superscripts (a,b,c) are significant difference (*p* < 0.05). TG: triglyceride, TC: total cholesterol.

## Data Availability

The data presented in this study are available on request from the corresponding author. The data are not publicly available due to ethical restriction.
